# The Role of Butyrylcholinesterase and Iron in the Regulation of Cholinergic Network and Cognitive Dysfunction in Alzheimer’s Disease Pathogenesis

**DOI:** 10.3390/ijms22042033

**Published:** 2021-02-18

**Authors:** Jacek Jasiecki, Monika Targońska, Bartosz Wasąg

**Affiliations:** 1 Department of Pharmaceutical Microbiology, Faculty of Pharmacy, Medical University of Gdańsk, 80-416 Gdańsk, Poland; 2 Department of Biology and Medical Genetics, Medical University of Gdańsk, 80-210 Gdańsk, Poland; monika.targonska@gumed.edu.pl (M.T.); bwasag@gumed.edu.pl (B.W.); 3 Laboratory of Clinical Genetics, University Clinical Centre, 80-952 Gdańsk, Poland

**Keywords:** Alzheimer’s disease, butyrylcholinesterase, pseudocholinesterase, iron, IRE, BChE, BuChE, neuroinflammation, amyloid

## Abstract

Alzheimer’s disease (AD), the most common form of dementia in elderly individuals, is marked by progressive neuron loss. Despite more than 100 years of research on AD, there is still no treatment to cure or prevent the disease. High levels of amyloid-β (Aβ) plaques and neurofibrillary tangles (NFTs) in the brain are neuropathological hallmarks of AD. However, based on postmortem analyses, up to 44% of individuals have been shown to have high Aβ deposits with no clinical signs, due to having a “cognitive reserve”. The biochemical mechanism explaining the prevention of cognitive impairment in the presence of Aβ plaques is still unknown. It seems that in addition to protein aggregation, neuroinflammatory changes associated with aging are present in AD brains that are correlated with a higher level of brain iron and oxidative stress. It has been shown that iron accumulates around amyloid plaques in AD mouse models and postmortem brain tissues of AD patients. Iron is required for essential brain functions, including oxidative metabolism, myelination, and neurotransmitter synthesis. However, an imbalance in brain iron homeostasis caused by aging underlies many neurodegenerative diseases. It has been proposed that high iron levels trigger an avalanche of events that push the progress of the disease, accelerating cognitive decline. Patients with increased amyloid plaques and iron are highly likely to develop dementia. Our observations indicate that the butyrylcholinesterase (BChE) level seems to be iron-dependent, and reports show that BChE produced by reactive astrocytes can make cognitive functions worse by accelerating the decay of acetylcholine in aging brains. Why, even when there is a genetic risk, do symptoms of the disease appear after many years? Here, we discuss the relationship between genetic factors, age-dependent iron tissue accumulation, and inflammation, focusing on AD.

## 1. Introduction

Alzheimer’s disease (AD) is a slowly progressive neurological disorder in which neurodegeneration is believed to begin 20–30 years before clinical onset [1]. Late-onset AD (LOAD) is clinically characterized by a progressive decline in memory and other cognitive functions, leading to the loss of the ability to perform everyday activities [2]. AD’s clinical expression correlates with synaptic damage accompanied by neuronal loss in the brain, particularly in the hippocampus and cerebral cortex [3,4,5,6]. The mainstay of the therapy approved to treat AD dementia is based on only a few drugs, cholinesterase inhibitors (ChEIs), that act by increasing neurotransmitters’ availability at synapses in the brain [7,8,9,10,11,12]. The therapy treats the final symptoms but does not prevent the underlying cause of the disease. The are many hypotheses regarding the primary cause of AD, including cholinergic neuron damage, the accumulation of proteins such as amyloid-β (Aβ) in plaques, hyperphosphorylated-tau in neurofibrillary tangles leading to massive loss of synapses, inflammation, the role of butyrylcholinesterase (BChE) in forming Aβ plaques, and oxidative stress. By definition, age is the most potent risk factor for LOAD [13,14,15,16].

However, the disease seems to be multifactorial, with environmental and genetic factors contributing to disease risk, manifestation, and progression [17]. The results from genome-wide association studies (GWAS) have shown that the ε4 allele of *APOE* is the most potent genetic risk factor for LOAD [18,19,20,21,22], followed by recently detected genetic risk factors that encode proteins involved in microglial function and inflammation, including triggering receptor expressed on myeloid cells-2 (TREM2) [23,24]. Many studies have shown that an imbalance between the production and clearance of amyloid-β (Aβ) forming amyloid plaques is probably a significant contributor to neurodegeneration and disease development [25]. TREM2, present on the microglia cell membrane, interacts with apolipoprotein E (ApoE) and clusterin (CLU), leading to the internalization of apolipoproteins linked with Aβ [26,27,28]. Apolipoprotein E (ApoE) is a protein mostly produced by astrocytes and microglia, and its function is to distribute cholesterol and lipids to neurons through binding to cell-surface ApoE receptors [29]. Additionally, ApoE forms complexes with Aβ and plays a role in the clearance and metabolism of Aβ [30]. The lipidation of ApoE and CLU enhances the eliminated Aβ process. ApoE ɛ4 indicates reduced lipidated status compared to other allelic forms, leading to ineffective clearance [26,31]. ApoE influences the propensity of Aβ to aggregate into fibrillar plaques, affecting the rate of Aβ clearance from the brain and the rate of conversion of Aβ monomers and oligomers to mature fibrils, as well as the influence on microglial activation [32,33]. 

Aβ refers to peptides derived from the amyloid precursor protein (APP) that vary in length from 36 to 43 amino acids and are a major component of the amyloid plaques found in AD brains [34,35,36]. Aβ40 is the most common, but Aβ42 is more fibrillogenic and thus more associated with AD [37,38,39]. It has been proposed that the neurotoxicity of the Aβ42 is linked to its aggregation state and interactions with metal ions such as iron, aluminum, copper, and zinc, which could play a crucial role in the release of reactive oxygen species (ROS) [36,40,41]. Accumulation of Aβ plaques is thought to initiate a pathogenic cascade that leads to synaptic dysfunction and neurodegeneration [42,43].

## 2. Neuroinflammation in AD

Postmortem analyses revealed that proteinopathy of AD caused by abnormal Aβ aggregation is observed in 30–40% of cognitively normal individuals [44,45,46,47]. However, only subjects with a build-up of both Aβ plaques and high iron levels are likely to develop dementia. It has been proposed that high iron levels trigger an avalanche of events that push the progress of the disease, accelerating cognitive decline [48]. Iron is significantly elevated in multiple cortical regions of AD brains, and its accumulation may cause neuronal loss, possibly by inducing oxidative stress and neurodegeneration by ferroptosis [49,50]. 

Iron serves as a cofactor in several biological processes, including transport of oxygen, electron transfer in cytochromes, ATP synthesis, regulation of protein expression cell growth, and differentiation [51,52]. Moreover, iron is involved in the synthesis of neurotransmitters and myelination, and it is bound in iron–sulfur clusters that are required for many enzymes in the brain. The brain is one of the most metabolically active organs in the body, and an adequate supply of iron is essential to meet its high energy requirements [53].

The pleiotropic function of iron comes from its ability to reverse transition between its two oxidation states, ferrous (Fe^2+^) and ferric (Fe^3+^). However, ferrous ions can react with a product of mitochondrial respiration, hydrogen peroxide, to generate hydroxyl free radicals in the Fenton reaction. Subsequently, free radicals increase ROS production and enhance oxidative stress, promoting lipid peroxidation that can induce cell death by a pathway, ferroptosis, and thus neurodegeneration. Iron is a toxic trace element. Therefore, it has to be circulated throughout the body, attached to its carrier proteins or chelators involved in iron uptake, storage, and export in peripheral tissues [54,55,56,57]. 

Ferroptosis is a type of programmed cell death that involves iron dysregulation, lipid peroxidation, and inflammation. Ferroptosis is initiated by depletion of glutathione peroxidase (GPX4), resulting in an accumulation of lipid ROS in cells, leading to oxidative cell death. Interestingly, GPX4 knock-out in mice revealed not only iron dysregulation, lipid peroxidation, and inflammation, but also signs of AD and neurodegeneration [49,58].

It has been hypothesized that brain iron elevation occurs after the formation of Aβ plaques and neurofibrillary tangles (NFTs), and iron accumulation causes toxicity and neurodegeneration by inducing oxidative stress or cell death via ferroptosis [59]. How brain iron can trigger neurodegeneration remains incompletely understood. Here, we describe the known mechanisms involved in neuroinflammatory processes in the brain, with the focus on iron and AD.

Dietary iron absorbed by the gut enterocytes is transported throughout the body in blood plasma, bound to the iron carrier protein transferrin (Tf) as Tf-bound iron (TBI) [60]. Usually, only 30% of Tf proteins carry iron, which gives a large buffering capacity in case of increment in iron plasma concentration. TBI circulating in the blood cannot enter the brain directly, and the transport is controlled in a multistep transcellular pathway by the blood–brain barrier (BBB). The BBB is formed by brain microvascular endothelial cells (BMVECs), astrocytes, and pericytes, forming tight junctions. Most brain iron is acquired by Tf receptor (TFR-1)-mediated endocytosis at the lumen BMVECs of brain capillaries [61,62]. When the acidic pH is reached in endosomes, iron is released from Tf, and endosomal ferri-reductase catalyzes the reduction of Fe^3+^ to Fe^2+^, enabling Fe^2+^ export into the cytosol from the endosome through divalent metal transporter-1 (DMT1) [63].

After crossing into the cytosol of the BMVEC, iron can be utilized in metabolic pathways, stored in the cytosolic and mitochondrial ferritin, or exported to the interstitial fluid in the brain via ferroportin (FPN) [64]. Ferritin is the cytosolic storage protein used to sequester iron in cells, keeping iron in a soluble, nontoxic but bioavailable form [65,66,67]. Both forms of iron, non-Tf-bound iron (NTBI) and TBI, have been identified in the brain interstitial fluid. However, NTBI is considered to be a physiologic form of iron, and the amount of TBI in the brain is thought to be 100 times less than levels circulating in plasma, whereas NTBI levels are high [68,69,70]. 

The brain comprises neurons and glial cells that include oligodendrocytes, astrocytes, ependymal cells, and microglia. These cells can uptake both Tf-bound and non-Tf-bound iron via two distinct pathways, via DMT-1 and TFR-1 receptors, from the interstitial fluid. Oligodendrocytes only acquire non-Tf-bound iron via the Tim-1 receptor [71,72,73,74]. Iron can only be exported by these cells through FPN, a receptor controlled by hepcidin [61,64].

One evident hallmark of neuroinflammation is the cells’ activation and increased acquisition of extracellular iron, causing the intracellular sequestration of iron in response to exogenous and endogenous danger signals. Such iron withdrawal plays a role in the brain to reduce the iron availability for bacteria, malignancies, and the endogenously produced ROS [75,76,77,78,79].

Microglia, which firstly respond to dangerous stimuli, are very long-lived myeloid immune cells of the central nervous system (CNS) (brain-resident macrophages), comprising up to 20% of the total brain glial cells. Their primary function is to search for and respond to danger signals by activation. M1 activation is proinflammatory and neurotoxic in response to the bacterial lipopolysaccharides, Aβ, interleukin 1 (IL-1), and ROS [80,81,82,83,84,85]. M1-activated microglia cells secrete proinflammatory cytokines such as tumor necrosis factor-alpha (TNF-α), interleukin-6 (IL-6), interleukin-1-beta (IL-1β), and interleukin-12 (IL-12) [86]. Microglia in the M1 state also produce nitric oxide synthase (iNOS), which produces nitric oxide that increases the toxic effects of glutamate and consequently potentiates *N*-methyl-d-aspartate (NMDA)-receptor-mediated neurotoxicity [87,88,89]. In the anti-inflammatory M2 state induced by interleukin-4 (IL-4) and interleukin-13 (IL-13), cells secrete anti-inflammatory cytokines such as interleukin-10 (IL-10) and transforming growth factor-beta (TGF-β). Danger signals, such as LPS, induce intracellular iron sequestration in cytosolic and mitochondrial ferritin in microglia and astrocytes to hide it from pathogens [90,91]. However, excess cytosolic iron can generate excessive ROS and the risk of microglial activation and inflammation of inflammasomes. NLRP3 inflammasomes are cytosolic multiprotein complexes abundantly expressed by microglia and astrocytes in response to dangerous stimuli like LPS, amyloid-β, iron-damaged mitochondria, and ROS [83]. The M1 state of microglia is activated by the NLRP3 inflammasome assembly with caspase-1 and triggers the release of IL-1β and IL-18 [92].

**Figure 1:**
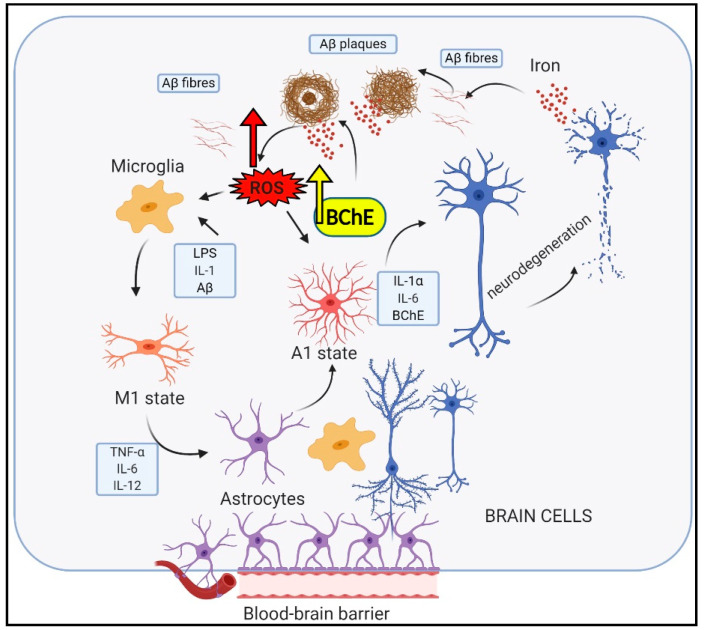
Neuroinflammation scheme. Danger signals, like reactive oxygen species (ROS) and LPS, are microglia activators and induce intracellular iron sequestration. LPS stimulates astrocytic hepcidin synthesis, which prevents iron efflux. LPS inhibits TREM-2 expressed on microglial cells, lowering AB clearance. Intracellular iron is bound to cellular ferritin but can dissociate and, via ROS, activate NLRP3 inflammasome, which triggers inflammation and microglial activation, M1 state. M1 microglial release of TNF-α, IL-1α, and C1q, which induce the phagocytosis of neurons and oligodendrocytes by A1 astrocytes, contributing to neurodegeneration and Alzheimer’s disease (AD). A1 astrocytes produce IL-1α, IL-6, and BChE.

M1-activated microglia, by secreting Il-1α, TNF, and C1q, convert trophic astrocytes to a reactive subtype, termed A1. Together, these cytokines are necessary and sufficient to alter astrocytic transcriptomes, inducing the neurotoxic A1 cells to kill healthy neurons and oligodendrocytes [93,94,95]. Nonactivated astrocytes are essential for CNS and perform many functions, including maintenance of extracellular ion balance; biochemical support of BMVECs; and regulation of homeostasis, metabolism, and synaptic transmission. Growing evidence reports the significant role of A1 astrocytes in various human neurodegenerative diseases, including Alzheimer’s, Huntington’s, and Parkinson’s diseases and multiple sclerosis. A1 astrocytes localize to Aβ plaques in AD. Astrocytes are the most abundant glial cells in the CNS and play a critical role in neuroinflammation and produce chemokines and cytokines, including interleukin-1-beta (IL-1β) and interleukin-6 (IL-6) [96]. Additionally, astrocytes also produce butyrylcholinesterase (BChE), the increased expression of which strongly correlates with the activation of astrocytes [97,98] (Figure 1).

## 3. Brain Iron and Aging

The concentration of iron varies significantly in different parts of the brain. Deposition of iron in the brain is positively correlated with age, with high concentrations in the frontal cortex, substantia nigra, basal ganglia, and hippocampus [99]. More iron is observed at the cellular level as a function of age in the microglia and astrocytes of the cortex, cerebellum, hippocampus, basal ganglia, and amygdala, which are particularly susceptible to the neuropathological changes that characterize AD [100,101,102]. Iron accumulation can stimulate the activation of these cells in the neuroinflammatory processes that contribute to AD. Iron levels in the brain rise with aging, and excess active iron can be toxic through various mechanisms, including oxidative stress and the promotion of lipid peroxidation that can induce cell death by a regulated cell death pathway, ferroptosis [49,58,103]. Even a delicate imbalance in brain iron is likely to have an adverse effect because, unlike serum transferrin (Tf), which is only 30–40% saturated with iron, brain Tf is 100% saturated and has a limited capacity for buffering excess iron [53,104,105].

## 4. Regulation of Iron Homeostasis 

Iron enters brain cells mainly via DMT-1 and TFR-1 [62,106,107]. Its metabolism is controlled by two regulatory systems, one that relies on the iron exporter FPN and its regulator, hepcidin, and the second that controls cellular iron levels through iron-regulatory proteins that bind iron-responsive elements in regulated messenger RNAs [108].

Hepcidin is a peptide hormone produced by hepatocytes, but only a small fraction of this peptide hormone crosses the blood–brain barrier (BBB) [79,109]. The brain biosynthesizes its own hepcidin by astrocytes and microglia in response to LPS and proinflammatory cytokines via the IL-6/STAT3 and SMAD4 pathways [110,111]. Hepcidin in the extracellular space inhibits iron export by binding to FPN and mediates FPN internalization and degradation in lysosomes [64]. In the CNS, iron sequestration by hepcidin may be beneficial and neuroprotective as it denies iron to pathogens, limiting infection and inflammation [79]. On the other hand, aging enhances the expression of hepcidin and LCN-2, increases the concentration of intracellular iron, and promotes inflammation via NLRP3 inflammasomes [84,112,113]. It seems that hepcidin brain secretion may have both beneficial and detrimental effects, depending on the cause of inflammation. 

Iron efflux via FPN is also controlled by APP [114,115]. APP is a transmembrane glycoprotein that plays many roles in the nervous system, including neuronal development, signaling, intracellular transport, maintenance of dendritic structure, and regulation of synapses [34,116,117,118,119]. Non-amyloidogenic α-secretase processing of APP generates sAPPα, which binds to FPN on the cell surface and stabilizes it, thus promoting neuronal iron efflux and decreased intraneuronal iron. On the other hand, amyloidogenic processing of APP impairs iron export by depleting FPN on the cell surface, thus increasing cellular iron that induces neurodegeneration. It is proposed that in this way, β-secretase processing of APP might indirectly promote ferroptosis [120,121,122]. Strikingly, a transgenic mouse model that has abundant Aβ deposition is not sufficient for synapse loss. However, β-secretase cleavage of APP by itself has been reported to cause synaptic and memory deficits [123].

Cellular iron homeostasis involves the coordination of iron uptake, storage, and efflux to ensure appropriate iron levels inside the cell and is controlled by the second mechanism at the post-transcriptional level. Brain iron homeostasis is regulated by a post-transcriptional gene expression regulation system composed of iron-regulatory proteins (IRPs), IRP1 and IRP2 and iron-responsive elements (IREs) that are present in mRNAs encoding for essential proteins of iron homeostasis. IREs are conserved mRNA motifs of 25–30 nucleotides located in the untranslated regions (UTRs) of mRNAs that can form a stem-loop [124]. Under high-cellular-iron conditions, the IRPs cannot bind the IREs because IRP1 assembles an iron–sulfur cluster, and it acts as a cytosolic aconitase, while IRP2 is degraded by the proteosome. Therefore, IRPs become IRE-binding proteins only in low concentrations of iron. Depending on the IRE position in the untranslated regions (UTRs) of mRNA, IRP binding regulates gene expression differentially. IRPs bound to IREs at the 5′UTR of mRNA inhibit translation initiation by preventing the recruitment of the small ribosomal subunit to the mRNA. The IRPs bound to IREs at the 3′UTR of mRNA decrease their turnover by preventing endonucleolytic cleavage and mRNA degradation. The IRP mRNA stabilization mechanism has not been fully understood yet for all 3′-IRE-containing mRNAs, such as DMT1, which only has a single 3’IRE and may require additional regulation factors [63,108,125]. In the case of a high iron concentration in cells, IRPs bind to the IRE of TFR1 and DMT1 mRNAs, decreasing their translation and resulting in lower cellular uptake of iron. In the opposite situation, when cellular iron levels are low, IRPs bind to the IRE at the 3′-UTR of TFR1 and DMT1 mRNAs, stabilizing them, increasing their cellular expression level, and thus increasing iron uptake. IRP–IRE interactions regulate the expression of the mRNAs encoding essential proteins for iron homeostasis, such as transferrin receptor 1 (TFR1), divalent metal transporter 1 (DMT-1), H-ferritin (Fth1), L-ferritin (Ftl), mitochondrial aconitase (Aco2), and ferroportin (FPN) [126].

Under low-iron conditions, IRP1 also binds to the IRE at the 5′-UTR of APP mRNA to repress APP translation [127,128]. A high iron load upregulates APP translation and increases the amyloidogenic processing of APP that generates Aβ peptide [85]. Monomeric Aβ reduces oxidative stress, inhibits Fe^3+^ reduction, and prevents lipid peroxidation and ferroptosis induced by Fe^2+^ [129,130,131].

## 5. Butyrylcholinesterase in AD

Butyrylcholinesterase (UniProt P06276), also known as plasma cholinesterase or pseudocholinesterase, is a serine hydrolase present in most tissues, with the highest levels in plasma and the liver [132]. Although the enzyme BChE and its coding gene were discovered years ago, still little is known about the regulation of the expression and its biological role, particularly in the central nervous system (CNS) [133,134]. BChE is expressed during mitosis in early embryonic development and promotes proliferation prior to differentiation [135].

BChE has a widespread distribution in the human body, and it serves as an inherent protector from damage caused by toxic compounds before they reach acetylcholinesterase (AChE) in synapses. BChE is found in glia and white matter in the brain, and it is involved, along with AChE, in cholinergic neurotransmission [136,137]. In the human brain, BChE is mainly expressed in glial cells, particularly astrocytes, in contrast to AChE, which is found in neurons. Nevertheless, BChE is also found in specific neurons, mainly localized in the amygdala, hippocampus, and thalamus [138,139]. The primary source of BChE in the CNS is non-neuron cells such as astrocytes and microglia, which also express nicotinic acetylcholine (ACh) receptors, indicating that BChE might play a regulatory role in the functional status of these cells via its ACh-hydrolyzing activity [140]. BChE was found in amyloid plaques and neurofibrillary tangles (NFTs), suggesting that the protein may be involved in AD’s pathogenesis [141,142,143,144,145]. Other researchers demonstrated that BChE might transform Aβ from an initially benign to an eventually malignant form [146]. 

**Figure 2:**
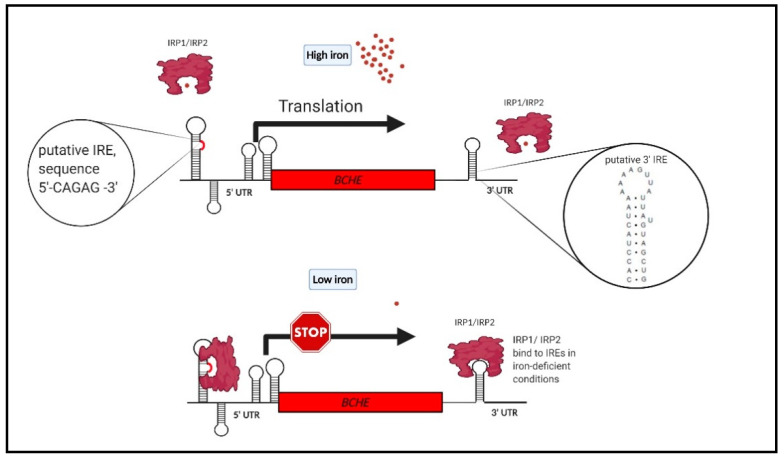
Proposed model of BChE expression regulation by iron in the activated glial cells. mRNA sequence analysis revealed putative IRE in the 3′-UTR of BChE (at low quality) and in the 5′-UTR. The Searching for IREs (SIREs) web bioinformatic program was used to predict the 3′-UTR iron-responsive elements in BCHE mRNA [154]. In 5′-UTR, only the consensus IRP binding sequence was found; however, since the expected structure of the 5′-UTR is complex [155], a stem-loop can be formed in a controlled process. Under conditions of high cellular iron, the IRPs cannot bind the IREs. IREs are conserved mRNA motifs. IRPs bound to IREs at the 5′-UTR of mRNA inhibit translation initiation by preventing ribosome binding to the mRNA. The IRPs bound to IREs at the 3′-UTR of mRNA decrease its turnover by preventing endonucleolytic cleavage and mRNA degradation. We suppose that the BChE expression can be positively regulated by iron in the 5′-UTR IRE element and in 3′-UTR by one stem-loop with additional regulator factors as a negative feedback mechanism. In order to validate the predicted IREs, the in vitro functionality has to be determined by competitive EMSA experiments.

**Figure 3:**
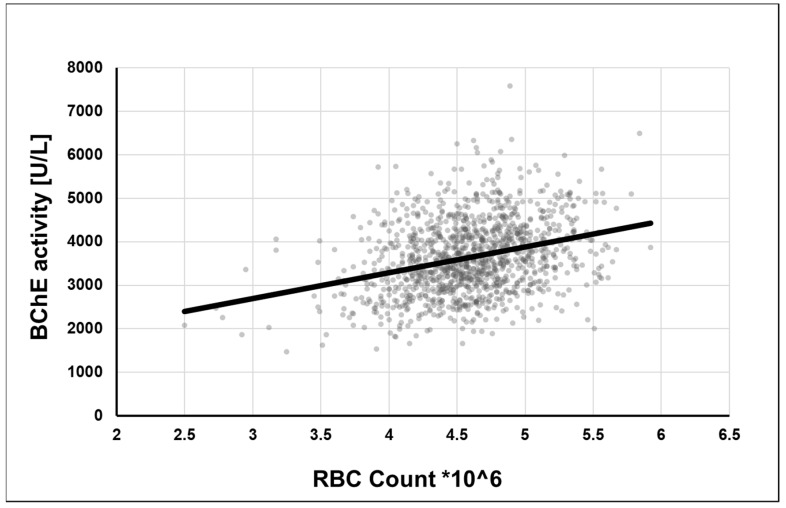
Interrelationship of peripheral serum butyrylcholinesterase (BChE) activity and red blood cell (RBC) count. The plasma BChE level shows a positive correlation with RBC count in a large cohort of 1191 individuals. The enzyme activity of a single person is represented by a grey dot. BChE activity was measured by Ellman’s reaction, as described earlier [156].

Interestingly, mRNA sequence analysis revealed putative IRE in 3′-UTR of BChE (at low quality) (Figure 2), suggesting that BChE can also be regulated by iron homeostasis. Under low iron concentration, IRP1/IRP2 proteins probably bind to stem-loop in 3′-UTR of BChE transcript and stabilize it by preventing degradation, thus increasing the BChE expression level. To support this hypothesis, we found a strong correlation between BChE activity and the red blood cell (RBC) count in a large cohort of 1200 individuals (not published previously, found the correlation of the old data) [147]. We observed that the level of the BChE activity is proportional to the RBC count (Figure 3). Since RBCs bind 65% of body iron, it seems that the BChE level is proportional to the body iron level [148]. The lifetime of RBCs is about 100–120 days, and the aging RBCs undergo eryptosis, programmed death. Much of the breakdown products are recirculated by the spleen and the liver. The iron is released into the plasma to be recirculated by Tf. Thus, the recirculated TBI is proportional to the RBC count. Since that the liver mainly produces serum BChE, the biosynthesis may be regulated similarly to TFR1 by IRPs. On the other hand, iron upregulates erythropoiesis levels by enhancing erythropoietin (EPO) synthesis by transcriptional activation. HIF-2α mediates the EPO gene’s transcriptional activation by binding to its hypoxia response element (HRE). Iron excess prevents binding of IRP1 to the IRE sequence at 5′-UTR of the HIF-2α and leads to prolonged translation of HIF-2α and thus increases the level of EPO [149,150,151,152,153]. Taken together, the BChE and the RBC levels can similarly reflect the concentration of cellular iron or TBI.

Strikingly, increased BChE activity is associated with A1 astrocytes in the AD brain. The expression of BChE is also increased in the hippocampus and temporal cortex of patients with AD, whereas the expression of AChE is reduced. 

## 6. Conclusions

Iron homeostasis becomes dysregulated during aging, leading to iron overload, which may promote neuroinflammation, protein aggregation, neurodegeneration, and AD development. Therefore, iron chelation and iron-targeted therapeutic strategies have been proposed as potential therapeutic strategies for neurodegenerative diseases [157]. Indeed, the anti-inflammatory iron chelator deferiprone is currently in phase 2 trials for AD and could soon become an FDA-approved iron chelator recommended for AD therapy [158]. Interestingly, conjugation of a cholinesterase inhibitor, such as galantamine, with the natural iron chelator lactoferrin was suggested as an AD therapy. Administration of iron chelates, like deferoxamine (DFO), significantly reverses changes due to iron overload. It has been shown that DFO inhibits Aβ toxicity via the modulation of Aβ–metal interactions, inhibits amyloidogenic APP processing, reduces the formation of ROS, and decreases Aβ oligomerization. DFO and other prospective metal chelators are promising drug candidates for managing neurodegeneration in the aging population [159,160,161].

Protein aggregation, neuroinflammation, and neuronal loss that lead to cognitive dysfunctions are neuropathological hallmarks of AD, and age-related iron dyshomeostasis can play a leading role in the processes. BChE produced by glial cells in response to proinflammatory signals and iron-dependent elevated levels of the BChE can make AD patients’ cognitive situation worse by accelerating acetylcholine decay in aging brains.

## Data Availability

Not applicable.
